# Ollier disease

**DOI:** 10.1186/1750-1172-1-37

**Published:** 2006-09-22

**Authors:** Caroline Silve, Harald Jüppner

**Affiliations:** 1 INSERM U. 773, Faculté de Médecine Xavier Bichat, 16 rue Henri Huchard, 75018 Paris, France; 2 Endocrine Unit, Department of Medicine, and Pediatric Neprology Unit, MassGeneral Hospital for Children, Massachusetts General Hospital and Harvard Medical School, Boston, MA 02114, USA

## Abstract

Enchondromas are common intraosseous, usually benign cartilaginous tumors, that develop in close proximity to growth plate cartilage. When multiple enchondromas are present, the condition is called enchondromatosis also known as Ollier disease (WHO terminology). The estimated prevalence of Ollier disease is 1/100,000. Clinical manifestations often appear in the first decade of life. Ollier disease is characterized by an asymmetric distribution of cartilage lesions and these can be extremely variable (in terms of size, number, location, evolution of enchondromas, age of onset and of diagnosis, requirement for surgery). Clinical problems caused by enchondromas include skeletal deformities, limb-length discrepancy, and the potential risk for malignant change to chondrosarcoma. The condition in which multiple enchondromatosis is associated with soft tissue hemangiomas is known as Maffucci syndrome. Until now both Ollier disease and Maffucci syndrome have only occurred in isolated patients and not familial. It remains uncertain whether the disorder is caused by a single gene defect or by combinations of (germ-line and/or somatic) mutations. The diagnosis is based on clinical and conventional radiological evaluations. Histological analysis has a limited role and is mainly used if malignancy is suspected. There is no medical treatment for enchondromatosis. Surgery is indicated in case of complications (pathological fractures, growth defect, malignant transformation). The prognosis for Ollier disease is difficult to assess. As is generally the case, forms with an early onset appear more severe. Enchondromas in Ollier disease present a risk of malignant transformation of enchondromas into chondrosarcomas.

## Disease name/synonyms

Ollier disease

Enchondromatosis

Multiple enchondromatosis

Dyschondroplasia

## Definition

Enchondromas are common benign usually asymptomatic cartilage tumors, which develop in the metaphyses and may become incorporated into the diaphyses of long tubular bones, in close proximity to growth plate cartilage [1-3]. Enchondromatosis (OMIM 166000) or Ollier disease (World Health Organization terminology) [4] is defined by the presence multiple enchondromas and characterized by an asymmetric distribution of cartilage lesions that can be extremely variable (in terms of size, number, location, evolution of enchondromas, age of onset and of diagnosis, requirement for surgery).

The condition in which multiple enchondromatosis is associated with soft tissue hemangiomas is known as Maffucci syndrome.

## Epidemiology

The estimated prevalence of Ollier disease is 1/100,000.

## Clinical description

Clinical manifestations in Ollier disease often appear in the first decade of life and usually start with the appearance of palpable bony masses on a finger or a toe, an asymetric shortening of an extremity with limping, osseous deformities associated or not with pathologic fractures [1-3]. Upon physical examination, enchondromas present on the extremities are usually visible as masses embedded within phalanges, metacarpal and metatarsal bones. Enchondromas frequently affect the long tubular bones, particularly the tibia, the femur, and/or the fibula; flat bones, especially the pelvis, can also be affected. The lesions may affect multiple bones and are usually asymetrically distributed, exclusively or predominantly affecting one side of the body. Affected bones are often shortened and deformed. Indeed, bone shortening may be the only clinical sign of the disease. These bone shortenings are often associated with bone bending and curving, and may lead to limitations in articular movement. Forearm deformities are frequently encountered and these are similar to those observed in hereditary multiple exostosis (HME). The trunc is usually not affected, except for rib enchondromas and scoliosis resulting from pelvis imbalance. In childhood, the lesions are subjected to pathologic fractures.

### Clinical forms

While enchondromatosis had been recognized for a long time, Ollier at the end of the 19th century (thus the name of Ollier's or Ollier disease to designate the condition) emphasized the asymetrical and random distribution of enchondromas. Some authors distinguish two subtypes of enchondromatosis, enchondromatosis and Ollier disease. The first form affects mostly men. It is characterized by enchondromas located mainly at the extremities and appears to be transmitted in an autosomal dominant fashion [5]. The second form affects mostly women. It is characterized by an unilateral distribution of enchondromas and appears sporadic. However, the basis for this classification into two forms is not supported by a thorough analysis of available clinical reports. In all instances, the association of multiple enchondromas with hemangiomas is referred to as Maffucci syndrome. Recently, a previously unreported form in which there is extensive involvement of the epiphyseal and metaphyseal regions of long bones of the lower extremity has been described [6].

## Radiography

**Figure 1:**
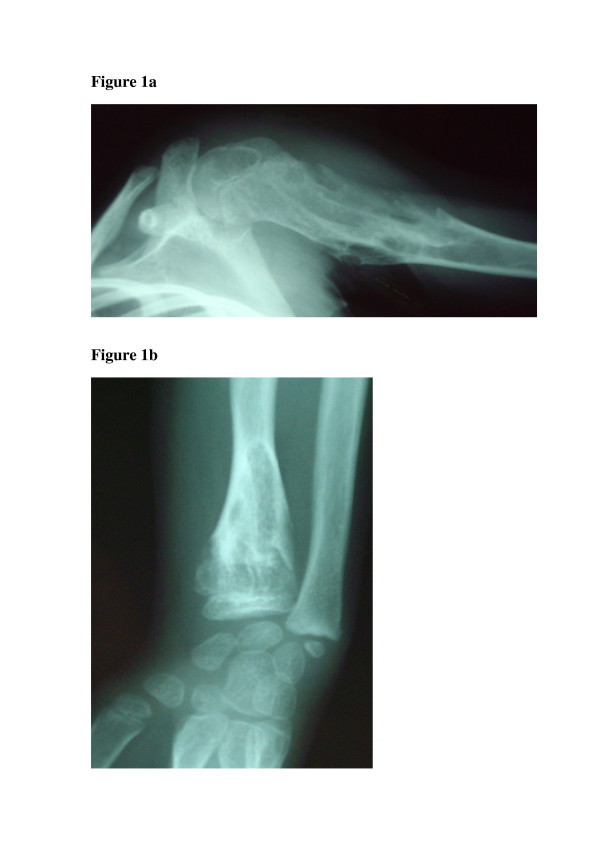
Roentgenographs showing enchondromas localized in the upper part of the humerus (fig 1a and lower part of the radius (fig 1b) of a girl 7 years of age affected with Ollier disease. Courtesy of Dr Fitoussi, Hôpital Robert Debré, Paris, France.

Enchondromas are rarely observed at birth, although the lesions are most likely already present. Roentgenograms typically show multiple, radiolucent, homogenous lesions with an oval or elongated shape and well defined slightly thickened bony margin [1-3]. The lesions and long bone axis run parallel (Figure 1). The lesions usually calcify with time and become diffusely punctated or stippled, a light trabeculation may be visible. Enchondromas are frequently assembled as clusters, thus resulting in the metaphyseal widening. When localized at the bone border, the enchondromas produce a typical notch-like image. A minor delay in bone age, on average 0.6 +/- 1.3 years, has been reported in children affected by Ollier disease [7].

Enchondromas are almost exclusively localized in the metaphysis of long bones and in the small bones of the hands and feet. They are initially localized close to the growth plate cartilage and then migrate progressively towards the diaphysis. The epiphyseal region next to an affected metaphysis may show irregularities [1,6].

Again, it is important to emphasize the irregular distribution of the lesions, which can be localized to one limb, or limited to one half of the body; however, even limited largely to one side of the body, one or two enchondromas are frequently present on the other side, in particular in the hand bones. If lesions are distributed over the entire body, one side is typically more affected. In the hands, the lesions almost never affect all metacarpal bones and phalanges.

Enchondromas result in severe growth abnormalities (more severe than those observed in multiple exostosis). Affected diaphysis are short and massively enlarged, and these may show bending close to the metaphysis. Ulnar shortening is usually more relevant than shortening of the radius; fingers often show irregular sizes. Signs of pathological fractures may be present.

Signs of malignant transformation should be looked for, as it is a major complication of enchondromatosis. These signs include cortical erosion, extension of the tumor into soft tissues, and irregularity or indistinctness of the surface of the tumor. Indeed, enchondromas tend to be well circumscribed, whereas chondrosarcomas show poor demarcation. The pattern of mineralization is also important in differentiating enchondromas from chondrosarcomas. As mentioned above, enchondromas tend to show a uniform pattern of mineralization, and the presence of unmineralized parts in the lesion is suspicious.

## Histopathology

Macroscopic examination of enchondromas usually shows multiple oval-shaped or round cartilaginous nodules in osseous portions of bone [1,2]. The individual nodules are limited at their periphery by woven or lamellar bone, and are separated from each other by intertrabecular marrow spaces. The cartilaginous tumor matrix is usually solid, with myxoid changes, which manifest as frayings of the matrix. Enchondromas are characterized by the presence of a striking heterogeneity and diversity in the degree of cellularity and chondrocyte phenotype. This heterogeneity depends to some extent on factors such as localization and the patient's age. In part due to this important cellular heterogeneity, the distinction between benign enchondromas and malignant chondrosarcomas by histochemical criteria is difficult. The histological criteria for malignancy that are used for conventional chondrosarcoma can not be used in Ollier disease because of the increased cellularity, and therefore the distinction between enchondroma and grade I chondrosarcoma in the context of enchondromatosis is extremely difficult or even impossible. The diagnosis therefore relies on the combination of radiographical (cortical destruction, soft tissue extension), clinical and histological criteria.

## Etiology and pathogenesis

Endochondral bone ossification is a highly regulated process, which requires the progression of undifferentiated mesenchymal cells into hypertrophic chondrocytes and the subsequent replacement of a cartilaginous matrix by mineralized bone [8,9]. Enchondromas develop in the metaphysis of long tubular bones in close proximity to the growth plate. Consequently, it was proposed that they result from abnormalities in signaling pathways controlling the proliferation and differentiation of chondrocytes, leading to the development of intraosseous cartilaginous foci.

### Genetics

Ollier disease – and Maffucci syndrome – are usually non-familial disorders [1-3], and both disorders thus appear to occur spontaneously and are not inherited. The irregular distribution of the lesions in Ollier disease strongly suggests that it is a disorder of endochondral bone formation that occurs due to a post-zygotic somatic mutation that results in mosaism. In two instances, enchondromatosis has been observed in the sons of fathers who presented with mild skeletal dysplasia but without evidence of enchondromas [5,10]. In one of these cases, a heterozygous mutation (R150C) in the PTH/PTHrP receptor (*PTHR1 *gene) was inherited from the father [10].

**Figure 2:**
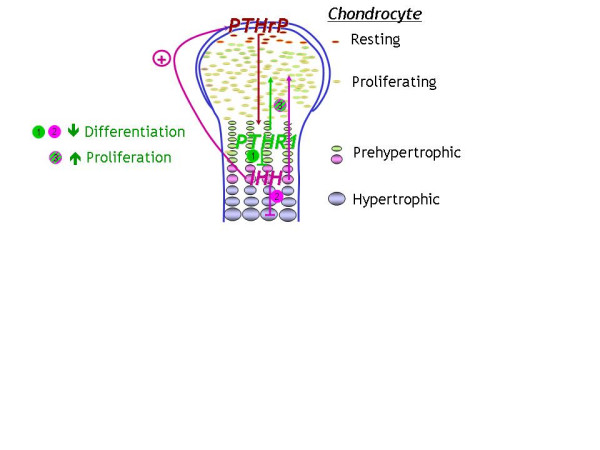
Importance of IHH and PTHrP signaling in the modulation of chondrocyte proliferation and differentiation during endochondral bone formation. PTHrP is synthesized by chondrocytes and perichondrial cells in the periarticular growth plate. PTHrP diffuses toward the prehypertrohic zone where it binds to and activates its receptor, the PTHR1, and thereby maintains chondrocyte proliferation and delays chondrocyte differentiation into pre-hypertrophic and hypertrophic chondrocytes. After chondrocytes stop proliferating at the transition from a proliferating into a hypertrophic phenotype, they synthesize IHH. IHH acts to indirectly increase the synthesis of PTHrP. IHH and PTHrP thus participate in a negative feed-back loop that serves to regulate the rate and synchrony of growth plate chondrocytes. Besides increasing PTHrP synthesis, IHH also stimulates, both directly and indirectly, chondrocyte proliferation and inhibits their terminal differentiation. Parathyroid hormone-related protein (PTHrP)  Indian Hedgehog (IHH)  Parathyroid hormone receptor 1 (PTHR1)

Parathyroid hormone-related protein (PTHrP) and Indian Hedgehog (IHH) acting on their respective receptors PTHR1 and PTCH1 exert a tightly coupled signaling relay, which is critical for the regulation of endochondral ossification (Figure 2). A mutant PTHR1 (R150C) was found to be expressed in the enchondromas from two of six unrelated patients with enchondromatosis [10]. The mutation was found on one parental allele in one patient and his father, who presented with atypical mild skeletal dysplasia, but not with enchondromatosis. However, neither the R150C mutation (26 tumors) nor any other mutation in the *PTHR1 *gene (11 patients) could be identified in another study, suggesting heterogeneity of the molecular defect(s) leading to enchondromatosis [11].

The mutant PTHR1 (R150C) seems to constitutively activate the PTHrP-dependent pathway, thus decreasing chondrocyte differentiation, thereby leading to the formation of enchondromas [10]. Consistent with this conclusion, transgenic mice expressing the mutant PTHR1 under the control of the collagen type II promoter develop tumors that are similar to those observed in human enchondromatosis. Because regulation of Ihh by PTHrP was found to be lost in these enchondromas, additional transgenic mice were generated that overexpress the Hedgehog (Hh) transcriptional regulator, Gli2. These mice develop ectopic cartilaginous islands similar to those observed in the mice expressing the mutant PTHR1. Thus, the Ihh signaling pathway as a whole seems to play a crucial role in the formation of enchondromatosis.

### Cytogenetics and molecular genetics

There are few cytogenetic reports of benign enchondromas, but there are no tumor-specific chromosomes or chromosomal regions associated with enchondromas, or chondrosarcomas [12-15].

Little is known about the molecular mechanisms involved in the malignant transformation from enchondromas to chondrosarcomas. Expression of PTHrP, the PTHR1, and their downstream partner Bcl2 may be correlated with the grade of malignancy in chondrosarcoma [16-19].

## Diagnostic methods

The diagnosis of Ollier disease is based on clinical and conventional radiological evaluations. Histological analysis has a limited role and is mainly used if malignancy is suspected. Additional investigations, such as scintigraphy, ultrasound, magnetic resonance imaging (MRI) are not useful for establishing the diagnosis. They are indicated for the evaluation and surveillance of lesions that become symptomatic (pain, increase in size).

## Differential diagnosis

Ollier disease must be differentiated from HME [1-3]. HME is an autosomal dominant disorder characterized by multiple bone tumors capped by cartilage, that occur mostly in the metaphyses of long bones. To establish the diagnosis of either disease, clinical and radiological criteria are used. The most important criterium to distinguish enchondromas from osteochondromas as seen in HME is the localization of bone lesions: osteochondromas are located at the bone surface and enchondromas are located in the center of bones, thus allowing radiographic distinction.

Other rare forms of chondromatosis, which include metachondromatosis, spondyloenchondroplasia and genochondromatosis type I and II, are described and have been well defined [1].

## Genetic counseling

Ollier disease – and Maffucci syndrome – are usually sporadic, non-familial disorders.

## Treatment

There is no medical treatment for Ollier disease. Surgery is indicated in case of complications (pathological fractures, growth defect, malignant transformation).

## Prognosis

The prognosis of Ollier disease is difficult to assess [1]. Patient with numerous lesions may have a better prognosis than patients with localized cartilaginous changes, which may induce major shortening of a lower extremity and thus limb asymmetry, especially if already present in a very young child. Similarly, early development of enchondromas in phalanges may lead to major finger deformities. As is generally the case, forms with an early onset appear more severe. Neural compressions are less frequently observed than in HME. Enchondromas in Ollier disease present a risk of malignant transformation of enchondromas into chondrosarcomas, which usually occurs in young adults, and thus at an earlier age than observed in patients with chondrosarcoma alone. The reported incidence of malignant transformation is variable and estimated to occur in 5–50% of the cases [3,20-22]. It is higher in Maffucci's syndrome, the prognosis of which is more severe than that of Ollier disease [1,2]. Association of Ollier disease with other tumors has been reported [1,23-25].

## Unresolved questions

• Although an identical heterozygous mutation in the *PTHR1 *gene has been identified in two unrelated patients with Ollier disease, this or other mutations in this gene were not identified in additional patients with this disorder. Although these studies need to be extended, they suggest that the cause of Ollier disease is heterogenous and raise the possibility that two (or more) genetic mutations are required to develop the disease. The development of enchondromas could thus be caused by a germline mutation associated with a somatic mosaic mutation. Furthermore, additional mutational events may underly progression from enchondromas to tumors.

• The molecular mechanisms involved in malignant transformation are unknown.

• The link, if any, between Ollier disease and Maffucci syndrome is unknown.

## References

[B1] Maroteaux P, Le Merrer M (2002). Les maladies osseuses de l'enfant.

[B2] Unni KK (2001). Cartilaginous lesions of bone. J Orthop Sci.

[B3] Whyte M (2003). Acquired Disorders of Cartilage and Bone.

[B4] Fletcher CDM, Unni K, Mertens F, (Ed) (2002). World Health Organization Classification of Tumors Pathology and genetics Tumors of Soft Tissue and Bone.

[B5] Halal F, Azouz EM (1991). Generalized enchondromatosis in a boy with only platyspondyly in the father. Am J Med Genet.

[B6] Gabos PG, Bowen JR (1998). Epiphyseal-metaphyseal enchondromatosis. A new clinical entity. J Bone Joint Surg Am.

[B7] Loder RT, Sundberg S, Gabriel K, Mehbod A, Meyer C (2004). Determination of bone age in children with cartilaginous dysplasia (multiple hereditary osteochondromatosis and Ollier's enchondromatosis). J Pediatr Orthop.

[B8] Kronenberg HM (2003). Developmental regulation of the growth plate. Nature.

[B9] Schipani E, Provot S (2003). PTHrP, PTH, and the PTH/PTHrP receptor in endochondral bone development. Birth Defects Res Part C Embryo Today.

[B10] Hopyan S, Gokgoz N, Poon R, Gensure RC, Yu C, Cole WG, Bell RS, Juppner H, Andrulis IL, Wunder JS, Alman BA (2002). A mutant PTH/PTHrP type I receptor in enchondromatosis. Nat Genet.

[B11] Rozeman LB, Sangiorgi L, Briaire-de Bruijn IH, Mainil-Varlet P, Bertoni F, Cleton-Jansen AM, Hogendoorn PC, Bovee JV (2004). Enchondromatosis (Ollier disease, Maffucci syndrome) is not caused by the PTHR1 mutation p.R150C. Hum Mutat.

[B12] Bovee JV, Cleton-Jansen AM, Kuipers-Dijkshoorn NJ, van den Broek LJ, Taminiau AH, Cornelisse CJ, Hogendoorn PC (1999). Loss of heterozygosity and DNA ploidy point to a diverging genetic mechanism in the origin of peripheral and central chondrosarcoma. Genes Chromosomes Cancer.

[B13] Bovee JV, van Roggen JF, Cleton-Jansen AM, Taminiau AH, van der Woude HJ, Hogendoorn PC (2000). Malignant progression in multiple enchondromatosis (Ollier's disease): an autopsy-based molecular genetic study. Hum Patho.

[B14] Sandberg AA (2004). Genetics of chondrosarcoma and related tumors. Curr Opin Oncol.

[B15] Sandberg AA, Bridge JA (2003). Updates on the cytogenetics and molecular genetics of bone and soft tissue tumors: chondrosarcoma and other cartilaginous neoplasms. Cancer Genet Cytogenet.

[B16] Amling M, Posl M, Hentz MW, Priemel M, Delling G (1998). PTHrP and Bcl-2: essential regulatory molecules in chondrocyte differentiation and chondrogenic tumors. Verh Dtsch Ges Pathol.

[B17] Bovee JV, van den Broek LJ, Cleton-Jansen AM, Hogendoorn PC (2000). Up-regulation of PTHrP and Bcl-2 expression characterizes the progression of osteochondroma towards peripheral chondrosarcoma and is a late event in central chondrosarcoma. Lab Invest.

[B18] Kunisada T, Moseley JM, Slavin JL, Martin TJ, Choong PF (2002). Co-expression of parathyroid hormone-related protein (PTHrP) and PTH/PTHrP receptor in cartilaginous tumours: a marker for malignancy?. Pathology.

[B19] Pateder DB, Gish MW, O'Keefe RJ, Hicks DG, Teot LA, Rosier RN (2002). Parathyroid hormone-related Peptide expression in cartilaginous tumors. Clin Orthop.

[B20] Rozeman LB, Hogendoorn PC, Bovee JV (2002). Diagnosis and prognosis of chondrosarcoma of bone. Expert Rev Mol Diagn.

[B21] Schaison F, Anract P, Coste F, De Pinieux G, Forest M, Tomeno B (1999). Chondrosarcoma secondary to multiple cartilage diseases. Study of 29 clinical cases and review of the literature. Rev Chir Orthop Reparatrice Appar Mot.

[B22] Schwartz HS, Zimmerman NB, Simon MA, Wroble RR, Millar EA, Bonfiglio M (1987). The malignant potential of enchondromatosis. J Bone Joint Surg Am.

[B23] Mahafza WS (2004). Multiple enchondromatosis Ollier's disease with two primary brain tumors. Saudi Med J.

[B24] Tamimi HK, Bolen JW (1984). Enchondromatosis (Ollier's disease) and ovarian juvenile granulosa cell tumor. Cancer.

[B25] Vaz RM, Turner C (1986). Ollier disease (enchondromatosis) associated with ovarian juvenile granulosa cell tumor and precocious pseudopuberty. J Pediatr.

